# Physical mechanisms driving the reversible aggregation of *Staphylococcus aureus* and response to antimicrobials

**DOI:** 10.1038/s41598-021-94457-1

**Published:** 2021-07-22

**Authors:** Céline Burel, Rémi Dreyfus, Laura Purevdorj-Gage

**Affiliations:** 1 Complex Assemblies of Soft Matter Laboratory (COMPASS), IRL 3254, CNRS-Solvay-University of Pennsylvania, CRTB, 350 George Patterson Boulevard, Bristol, PA 19007 USA; 2 Solvay, Novecare CRTB, Bristol, PA 19007 USA

**Keywords:** Antimicrobials, Bacteria, Colloids

## Abstract

Formation of non-sessile, auto-aggregated cells of *Staphylococcus aureus* contributes to surface colonization and biofilm formation, hence play a major role in the early establishment of infection and in tolerance to antimicrobials. Understanding the mechanism of aggregation and the impact of aggregation on the activity of antimicrobials is crucial in achieving a better control of this important pathogen. Previously linked to biological phenomena, physical interactions leading to *S. aureus* cellular aggregation and its protective features against antimicrobials remain unraveled. Herein, in-vitro experiments coupled with XDLVO simulations reveal that suspensions of *S. aureus* cells exhibit rapid, reversible aggregation (> 70%) in part controlled by the interplay between cellular hydrophobicity, surface potential and extracellular proteins. Changing pH and salt concentration in the extracellular media modulated the cellular surface potential but not the hydrophobicity which remained consistent despite these variations. A decrease in net cellular negative surface potential achieved by decreasing pH or increasing salt concentrations, caused attractive forces such as the hydrophobic and cell–protein interactions to prevail, favoring immediate aggregation. The aggregation significantly increased the tolerance of *S. aureus* cells to quaternary ammonium compounds (QAC). The well-dispersed cell population was completely inactivated within 30 s whereas its aggregated counterpart required more than 10 min.

## Introduction

The Gram-positive *S. aureus* is an opportunistic pathogen able to thrive in a wide range of environments such as on dry inanimate surfaces, in body fluids or on human skin^1^. Traditionally, *S. aureus* cells have been studied as free-floating planktonic organisms but lately, multicellular communities of *S. aureus* forming biofilms have attracted more attention due to their increased resistance to antimicrobials^2–4^. Recently, autoaggregation has been identified to be among the first steps in building a biofilm^5^. In contrast with biofilms, bacteria cell aggregates are not necessarily surface attached but are free-floating. They are also generally smaller than typical biofilms, and they do not form the mushroom-like towers that have been observed with in vitro biofilm growth^2^. A deeper understanding of the mechanisms underlying the aggregation of *S. aureus* cells and the impact of aggregation on the activity of antimicrobials will facilitate the development of novel disinfection and therapeutic strategies. Until now, the mechanism of aggregation in bacteria has mostly relied on biological phenomena such as quorum sensing, stress response, predation pressure or homotypic interactions between the bacteria surface and/or exogenous host proteins and molecules^5–7^. A different, yet complementary approach consists in viewing the suspensions of bacterial cells as colloids, and in applying principles of soft condensed matter physics to predict the behavior of living cells^8,9^. *S. aureus* is a non-motile, 1-micron spherical, negatively charged bacterium that closely resembles colloidal suspensions of particles when cultivated in a liquid medium. Destabilization of colloids and formation of aggregates can be caused by surface-surface attractive forces such as hydrophobic^10^ or oppositely charged^11^; depletion interactions^9,12^ and surface potential variations^13^. In this work, we demonstrate that planktonic suspension of *S. aureus* cells exhibit rapid, reversible aggregation in part driven by the bacteria surface potential and hydrophobicity. In our experiment the bacteria surface potential was governed by the pH and salt concentration of the extracellular medium. Bacteria cell aggregation was emphasized at low net cellular surface potential and by the presence of proteins. The consequence of such aggregation was studied in terms of bacterial tolerance to a common disinfectant, quaternary ammonium compound (QAC) and it was demonstrated that a population containing a greater fraction of aggregated cells was more than 20 times harder to kill than the one with fewer aggregates.

## Results and discussion

**Figure 1:**
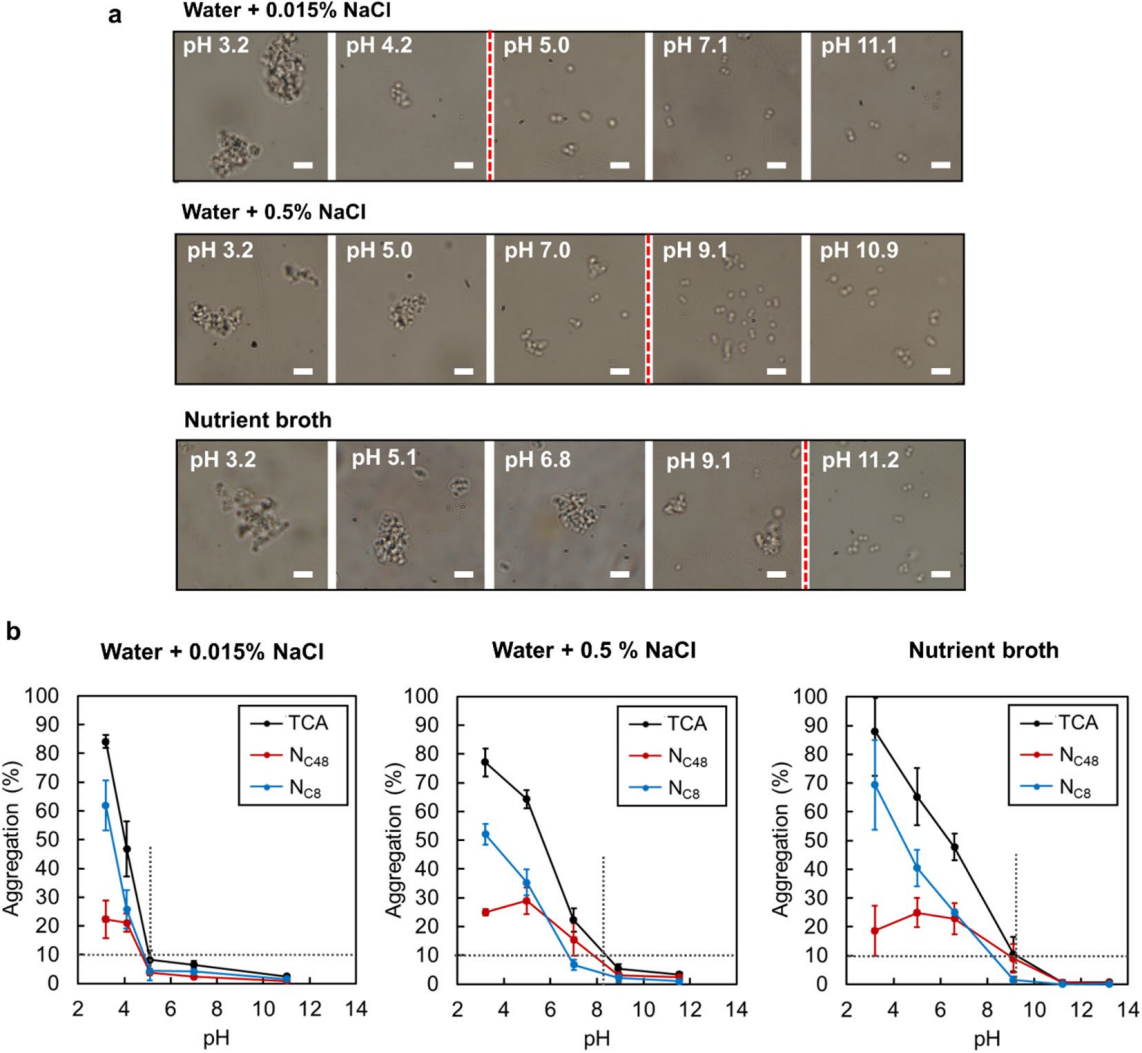
*S. aureus* cells aggregate when pH is decreased and salt or protein concentrations are increased. (**a**) Aggregation of *S. aureus* cells as a function of pH in water with 0.015% and 0.5% of NaCl and in Nutrient broth. Each scale bar is 4 µm. (**b**) Quantification of *S. aureus* cells aggregation as a function of pH in water with 0.015% and 0.5% of NaCl and in Nutrient broth. TCA is the total percent of cells aggregated, N_C48_ is the percent of cells in aggregates of sizes between 4 and 8 µm and N_C8_ is the percent of cells in aggregates larger than 8 µm. The crossover of the two dashed lines indicates the value of pH_Agg_.

To elucidate if physical forces can cause *S. aureus* aggregation, hydrophobicity and aggregation behavior of *S. aureus* suspended in water with low (0.015%) and high (0.5%) concentrations of salt (NaCl) and in Nutrient broth was studied as a function of pH (Fig. 1). Nutrient broth is a proteinaceous medium used in many standardized microbiology tests. A 0.015% (2.6 mM) NaCl solution was selected to match the salt concentration of Nutrient broth and 0.5% NaCl (85.6 mM) was selected because it is used in other standardized media including AOAC Nutrient broth in addition to being a concentration relevant to physiological conditions. In each media, multiple bright-field optical microscopy images of the bacterial suspensions were analyzed at several pH values (“Materials and methods” section). Overall, *S. aureus* aggregation was observed to be pH-dependent in all three media. The aggregation occurred rapidly, in a matter of 15 s and the overall size of the aggregates noticeably increased as pH decreased (Fig. 1a). The total percent of cells aggregated (“TCA”) was evaluated based on the percentage of cells that were found in aggregates of sizes larger than 4 µm (Fig. 1b). An arbitrarily set pH, denoted as pH_Agg_, was defined as the threshold pH whereat 10 or more percent of the cell population were aggregated. The pH_Agg_ values were measured at approximately 4.8, 8.0 and 9.1 in water at low and high NaCl concentrations, and in Nutrient broth, respectively. As pH dropped below the pH_Agg_ value, a sharp increase in the overall TCA populations was observed in each medium, reaching up to 85% in water at low NaCl concentrations, 75% in water with 0.5% NaCl and 88% in Nutrient broth. The TCA population was further subdivided into two groups based on the cross-areal diameter of individual aggregates: percent of cells in aggregates of sizes between 4 and 8 µm (“N_C48_”) and percent of cells in aggregates larger than 8 µm (“N_C8_”). At pH < pH_Agg_, N_C48_ increases at a moderate rate to the reach maximum values of 20–29% whereas N_C8_ continuously increased, leading to the final values of 62%, 52% and 69% for water at low and high salt concentrations and for Nutrient broth, respectively. These observations could be explained by the formation of new aggregates in combination with the growth of already formed aggregates as pH dropped. Further analyses revealed that, in all three media, aggregates of sizes larger than 8 µm grew both in size and in number at pH below the pH_Agg_ value (see Fig. S1 in the Supplements). In summary, when salt or proteins were present at physiologically relevant concentrations, the aggregation of *S. aureus* was enhanced. Indeed, the pH_Agg_ values shifted towards greater values along with more than 25% of the cell population existing in aggregates of at least 8 µm in size at larger pH values (pH 5 and 6.5 for 0.5% NaCl and Nutrient broth as compared to pH 4.2 in 0.015% NaCl).

**Figure 2:**
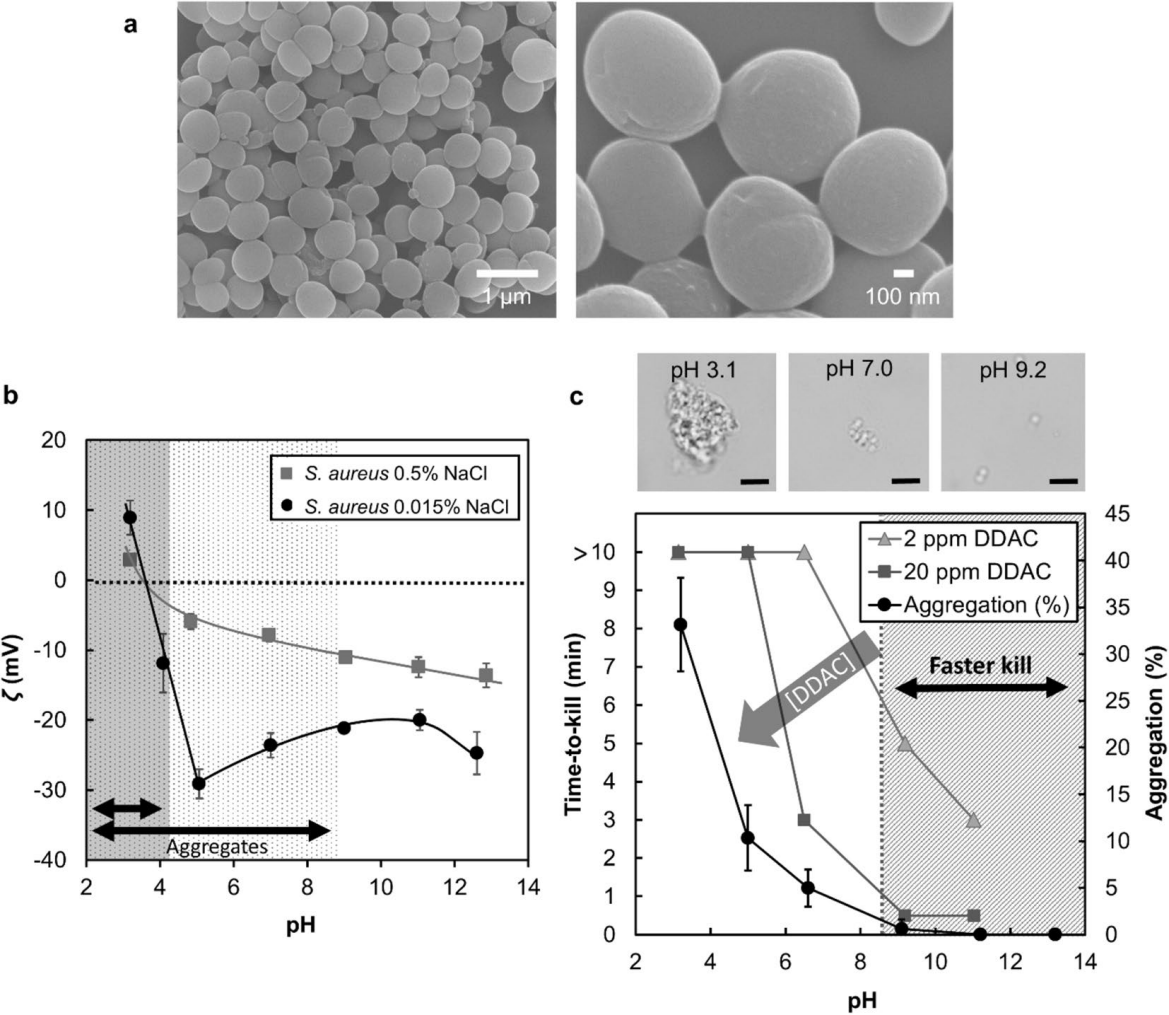
The aggregation of *S. aureus* depends on the potential of the cells and increases the bacterial tolerance to QACs. (**a**) SEM images of clustered *S. aureus* cells cultivated overnight in Nutrient broth (pH 7) reveal the absence of polymers or proteins in the cell clusters. (**b**) Surface potential of *S. aureus* in water with 0.015% and 0.5% of NaCl as a function of pH. (**c**) *S. aureus* time-to-kill as a function of DDAC concentration and pH (each datum point corresponds to three determinations with identical results) and simulated *S. aureus* (7 log_10_CFU/mL) aggregation as a function of pH. Optical microscopy images of *S. aureus* (7 log_10_CFU/mL) aggregates observed at 3 different pH values. Each scale bar is 4 µm.

In this work, the aggregation of *S. aureus* occurred in a matter of seconds upon change of the extracellular pH and salt content. Additionally, SEM imaging of cells grown in Nutrient broth at pH ~ 7 (“Materials and methods” section) did not reveal external web-like polymers or protein matrices surrounding the bacterial cells (see Fig. 2a). These observations suggest that the mechanism of aggregation in *S. aureus* may not derive from changes in cellular metabolism or from de-novo synthesis of adhesive factors (both requiring a longer time for gene expression and protein synthesis) as previously thought by Haaber et al^6^. Since the overall surface potential of bacteria is pH- and salt-dependent^14^, it is possible that the rapid aggregation observed in *S. aureus* could, in fact, be due to changes in the bacterial surface potential. We measured the surface potential of *S. aureus* as a function of pH at 0.015% and 0.5% NaCl. Results presented in Fig. 2b show that the absolute surface potential of *S. aureus* becomes less negative as pH decreases. Indeed, the zeta potential (ζ) of bacteria dispersed in water with 0.015% NaCl increases from − 25 mV (7 < pH < 11) to + 10 mV (pH 3). By decreasing the net surface potential, the electrostatic repulsion between the cells also decreased thereby causing aggregation. The addition of 0.5% of NaCl shifted the entire set of |ζ| values upwards on the zeta potential graph, indicating an overall decrease in the surface potential across the entire pH range studied. This behavior is characteristic of charge screening by salt in colloidal suspensions, as reported by Leckband and Israelachvili^13^ and resulted in a greater aggregation in water with 0.5% of NaCl as compared to 0.015% NaCl (Fig. 1b). In addition to surface potential, hydrophobic interactions can play an important role in cell–cell aggregation^13^. The hydrophobicity of *S. aureus* cells was measured^15^ and the results presented in Fig. S2 in Supplements showed the *S. aureus* strain used herein to be hydrophobic (in alignment with the results of Reifsteck et al.)^10^ regardless of the extracellular pH or salt content. Simulations of *S. aureus* aggregation using an extended DLVO model^16–19^ (XDLVO, see Fig. S3 in Supplements) revealed that without this hydrophobic property (modeled by attractive hydrophobic forces^13^), cells carrying a strong negative potential at pH 5–7 (below pH_Agg_), would not aggregate in water with 0.5% of NaCl. Indeed, across the pH range 3–12, the hydrophobic attractive forces are about twice as large as the *Van Der Waals* attractive forces. In Nutrient broth, the surface potential of bacteria is expected to be similar to the surface potential of bacteria dispersed in water containing a matching salt concentration of 0.015% NaCl. However, as compared to water with 0.015% NaCl, the aggregation of *S. aureus* was considerably enhanced in Nutrient broth (pH_Agg_ is shifted from 4.8 to 9.1). While Nutrient broth is a complex medium, proteins such as the ones it contains have already been reported to promote cell aggregation via several mechanisms^5,13^ including depletion and bacterial cells bridging. In our study, the two latter potential mechanisms could have been enhanced at low pH due to decrease in the net surface potential and aggregation of proteins contained in Nutrient broth (see Fig. S4 in Supplements).

The consequence of *S. aureus* cell aggregation was studied in relation to bacterial tolerance to QACs, which are broad-spectrum antimicrobials^20,21^ commonly used in disinfectant and sanitizer formulations. QACs are membrane-active agents comprising a positively-charged head which electrostatically interacts with the negatively charged bacteria membrane, and a hydrophobic tail which can penetrate into the membrane ultimately leading to membrane structural damages and bacteria lysis^20^. Suspensions of cells (6.7 log_10_CFU mL^−1^) in Nutrient broth were adjusted to pH values between 3 and 11. Then, each sample was challenged with either 20 or 2 ppm of Didecyldimethylammonium chloride (DDAC) for 30 s, 1, 2, 5 and 10 min (“Materials and methods” section). The minimum time required to inactivate the entire cell population (time-to-kill) was plotted as a function of pH. Results presented in Fig. 2c show that for both DDAC concentrations, the time-to-kill values decrease with increasing pH with the shortest time-to-kill values observed at pH 9 and 11 (see Tables S1 and S2 in Supplements). Two parts per million (ppm) of DDAC at pH 9 were sufficient to achieve a complete kill within 5 min of exposure time. Increasing both DDAC concentration and pH drastically reduced the time-to-kill. Indeed, we report a complete deactivation of *S. aureus* within seconds (30 s) of exposure to 20 ppm of DDAC at pH 9, compared to 3 min being required at pH 6.5. As pH is decreased from 7 to 3.2, the prediction of the aggregation for 6.7 log_10_CFU/mL yield aggregation of 35% of the cell population (see Fig. S5 in Supplements). Overall, *S. aureus* cells are less sensitive to DDAC when the bacterial surface is less negatively charged and the cell population is more aggregated. Interestingly, a tenfold increase in the DDAC concentration reduced the time-to-kill from more than 10 min to 3 min at pH 6.5 whereas a diminution of the time-to-kill was not observed at lower pH values (pH 5 and 3). As previously shown, when pH decreased, the proportion of aggregated cells increased, with larger and more numerous aggregates forming at the lowest pH values. Additionally, in low NaCl conditions such as found in Nutrient broth or water with 0.015% NaCl, the bacterial surface potential measured at pH 7 and pH 9 (Fig. 2b) were comparable (ζ ~ − 22 mV); whereas the aggregation of cells was greater at pH 7 than it was at pH 9. Thus, the superior antimicrobial efficacy observed with DDAC at pH 9 as compared to pH 7 cannot be attributed to the difference in the electrostatic attraction between negatively-charged bacteria and positively-charged QACs. We therefore, speculate that the increased tolerance to QACs observed at low pH values were likely driven by the physical barrier provided by cellular aggregation.

**Figure 3:**
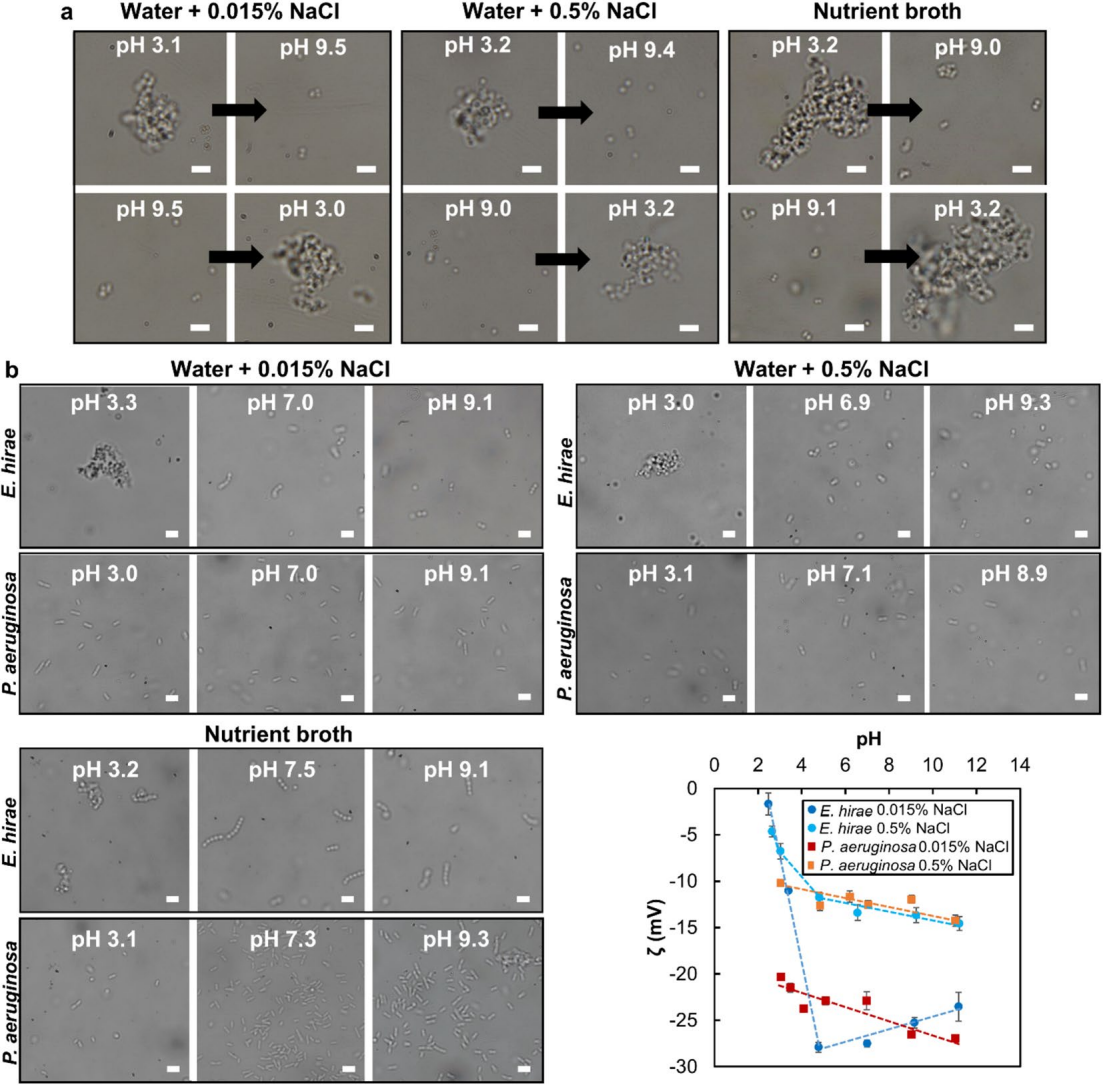
Cellular aggregation is reversible and specific to bacteria with a lower cell potential. (**a**) Reversibility of *S. aureus* aggregation in water with 0.015% and 0.5% NaCl and in Nutrient broth. (**b**) Aggregation behavior of *E. hirae* and *P. aeruginosa* in water with 0.015% and 0.5% NaCl and in Nutrient broth as a function of pH. Each scale bar is 4 µm. Surface potential of *P. aeruginosa* and *E. hirae* suspended in water with 0.015% and 0.5% NaCl. The dotted lines are to provide visual guidance. *E. hirae* aggregate at low pH (lower cell potential) while *P. aeruginosa* does not.

Bacterial aggregates including sessile biofilms often rely on external shear forces or secretion of enzymes to disperse^2,22^. To determine whether the aggregation in *S. aureus* is reversible, we imaged aliquots of *S. aureus* cells at pH adjusted to 3, and then raised to 9. The process was repeated in a different set of identical samples starting from pH 9 and then lowered to pH 3. Results presented in Fig. 3a show the aggregation to be reversible in all three media studied. Indeed, aggregates formed at pH 3 rapidly dispersed when the pH was raised to 9. Similarly, the singlet cells observed at pH 9 quickly aggregated when the pH was lowered to 3. This behavior suggests that in low pH ranges, the net surface potential of bacteria is small enough such that long range electrostatic interactions are overpassed by attractive interactions, thus inducing aggregation. Irreversible aggregation occurs when bacteria get extremely close to each other^13^. The observed reversible aggregation can be explained by the presence of a short range (~ 0.5 nm) repulsive interaction^17^ between the bacteria, which precludes any irreversible aggregation due to *Van der Waals* interactions. We have included such a repulsion in our XDLVO model in water with NaCl (see the Supplements for more details). Standard disinfection tests such as developed by the EPA^23^ and the “AOAC use dilution test”^24^ require the complete kill of test bacterial population within 10 min of exposure in proteinaceous media and are conducted as a qualitative assay based on turbidity similar to the methodology applied in this study. To meet such demanding standards, industrial and healthcare disinfectants often formulated at neutral pH (considered as an optimal pH for QACs performance) require the use of several thousands of ppm of QACs. Since aggregation of *S. aureus* is reversible with increasing pH, a change in formulation pH would be a quite simple solution to help inactivate this bacterium more efficiently, with lower usage of biocidal actives.

To investigate whether the surface potential-mediated aggregation of *S. aureus* is a general and observable trait in other species of bacteria, we imaged *Enterococcus hirae* (Gram positive, non-motile) and *Pseudomonas aeruginosa* (Gram negative, motile) suspended in Nutrient broth and in water with 0.015% and 0.5% NaCl, each adjusted to pH 3, 7 and 9. The results, presented in Fig. 3b, indicate that at pH 3 *E. hirae* cells aggregated whereas *P. aeruginosa* cells did not in all three media. The aggregation of *E. hirae* occurred within a few minutes, which was slower than that of *S. aureus*. The bacterial surface potential was determined as a function of pH for both bacteria dispersed in water with 0.015% and 0.5% NaCl. The results presented in Fig. 3b show that the net surface potential in *E. hirae* decreased significantly at lower pH values. On the contrary, the surface potential in *P. aeruginosa* decreases to a much lesser extent within the pH range of 2 to 12, confirming the results reported by Shephard et al^25^. Interestingly, in Nutrient broth, *P. aeruginosa* did not aggregate at low pH, which could be due to a stronger electrostatic repulsion between the bacteria overcoming the attractive forces mediated by proteins. Indeed, as compared to *E. hirae*, the net surface potential of *P. aeruginosa* is higher at pH below 4. These results confirm that a significant reduction in the bacterial net surface potential (< 11 mV) is necessary for cellular aggregation to occur in Nutrient broth.

In summary, the current research determined that short-time scale cellular aggregation of *S. aureus* is dependent on cell surface potential, which is governed by extracellular pH and salt concentration. At a higher pH, the strongly negatively charged cells are fully dispersed, whereas at a lower pH, cells with a lower net surface charge aggregate. The aggregation is amplified by the addition of salt due to cell surface charge screening, or proteins due to bacteria bridging or depletion. Our experiments revealed that the intrinsic hydrophobicity of *S. aureus* is an important bacteria feature contributing to cellular aggregation. *S. aureus* cellular hydrophobicity remained consistent despite variations in pH and salt concentrations. Additionally, our XDLVO model estimated that the hydrophobic interactions are about 2 times larger than the *Van Der Waals* attractive interactions. Upon decrease in net cellular negative potential, the attractive hydrophobic interactions prevail over the electrostatic repulsive interactions, hence leading to immediate aggregation. A similar surface potential driven aggregation behavior observed with *E. hirae* but not with *P. aeruginosa* suggests that bacteria with a lower net surface potential are more susceptible to aggregation. The impact of aggregation on the resistance of *S. aureus* to QACs is significant. The time-to-kill is 20 fold greater at low pH when aggregates are formed as compared to high pH where cells are dispersed. This result is in agreement with previous reports^6^ suggesting that bacterial aggregation provides a microenvironment in which innermost cells are protected from antimicrobial agents. Interestingly, *S. aureus* is known to acidify its extracellular medium as it multiplies in a closed batch system^26^. Moreover, *S. aureus* is often found on host skin^7^ which displays a slightly acidic pH when intact. A low pH environment might dissuade the growth of many organisms but may favor survival and formation of auto-aggregated populations of *S. aureus* cells that are self-protected. This protective phenotype combined with maintained mobility afforded by non-sessile aggregation, is likely to enhance survival and successful colonization of *S. aureus* under various environmental conditions. The reversible, surface potential-dependent nature of aggregation in *S. aureus* along other similarly charged bacteria could provide guidance to a design of more effective disinfectants and sanitizers.

This paper described aggregation behavior in *S. aureus* which was previously grown in the conditions required by standard disinfection test methods. Additional work is necessary to determine if similar aggregation mechanisms are applicable to other strains and growth conditions. Indeed, parameters such as bacterial strain, growth phase (exponential vs stationary), growth temperature and stock culture storage temperature (fridge vs room temperature) can impact expression profile of membrane and cell wall associated proteins. These changes in protein expressions can, in turn, affect bacterial surface charge and hydrophobicity^10^ ultimately impacting the forces driving the aggregation of *S. aureus*. Future investigations on these environmental variables will provide guidance on how bacterial aggregation impacts disinfection in both laboratory and real-world settings.

## Materials and methods

### Microbial strains

All bacterial strains were purchased from the American type culture collection (Manassas, VA, http://www.atcc.gov. *Staphylococcus aureus* (ATCC 6538), *Pseudomonas aeruginosa* (ATCC 15442) and *Enterococcus hirae* (ATCC 10541) cultures were maintained in a 40% (v/v) glycerol solution at − 80 °C.

### Bacteria cell culture and plate counting

Fresh cultures of *Staphylococcus aureus*, *Enterococcus hirae* and *Pseudomonas aeruginosa* were prepared by inoculating 35 mL Nutrient Broth (General Laboratory Products Inc.) with a single colony isolated on a Tryptic Soy Agar (TSA, Hardy Diagnostics) plate stored at 4ºC. For *S. aureus*, the same streak plate and Nutrient broth batch were used for all experiments. We observed a standard error in pH_Agg_ (± 0.6 pH unit) with varying batches of streak plate and Nutrient broth. The *S. aureus* liquid cultures were then incubated overnight at 35 °C while shaking at 150 rpm (incubating Minishaker 12620-942, VWR International). The *E. hirae* and *P. aeruginosa* liquid cultures were incubated overnight at 37 °C while shaking at 150 rpm. For *S. aureus*, the concentration of the overnight bacterial cultures was determined by serial dilution and plating on TSA plates. Colony forming units of bacteria per mL (CFU mL^−1^) were counted after 24 h of incubation at 35 °C. The final bacterial concentration in the overnight culture was about 8 log_10_CFU mL^−1^ for *S. aureus* (OD_600 nm_ ~ 0.3). The concentrations of *E. hirae* and *P. aeruginosa,* were ~ 7.3 (Nutrient Broth)/8.1(water with NaCl) and ~ 9.5 log_10_CFU mL^−1^, respectively.

### Aggregation study

#### In water with 0.015% NaCl

The overnight culture was centrifuged twice at 10,000 rpm for 10 min. The resulting bacterial pellets were re-dispersed in ultrapure water with 0.015% NaCl (BioXtra) at pH 7. The final OD_600nm_ of the bacterial dispersion was 0.3.

#### In water with 0.5% NaCl

The overnight culture was centrifuged twice at 10,000 rpm for 10 min. The resulting bacterial pellets were re-dispersed in ultrapure water containing 0.5% NaCl (BioXtra) at pH 7. The final OD_600nm_ of the bacterial dispersion was 0.3.

#### In nutrient broth

The overnight culture (OD_600nm_ = 0.3) was used without further dilution or wash step to mimic the culture preparation methods recommended by standard disinfection tests.

For all three media types (water with 0.015% and 0.5% NaCl and Nutrient broth), the final bacterial dispersions were aliquoted into 4 mL samples and were adjusted to appropriate pH levels using either hydrochloric acid or sodium hydroxide. A gentle manual shake was performed to homogenize each sample before analysis (taking place only a few minutes after pH adjustment). Optical imaging and zeta potential measurements were carried out using the same overnight culture. All experiments were performed in triplicates using three independent overnight cultures.

### Optical microscope imaging

For the hydrophobicity test, the optical microscopy images were acquired on a Leica TCS SP8 inverted confocal microscope in bright field and transmission mode. All the other images were acquired on an Olympus microscope in bright field and transmission mode. Images were taken with a 100 × objective oil (NA 1.4). The camera used was a Sony α Nex-7 color camera.

### Bacteria counts and statistical analysis

For the image analysis, a 4 µm size threshold was set based on the fact that *S. aureus* cells typically form singlets, duplets, triplets and quadruplets (made of 4 one-micron cells in a clusters) whose sizes are below 4 µm. Bacteria were counted manually for three independent replicate experiments. Approximately 2000 bacterial cells were counted for each replicate experiment and for each pH value, leading to a total of more than 6000 bacterial cells for each data points. The number of bacteria aggregated in all three replicate experiments were averaged and the standard deviation of the average was used for the error bars (error = Stdv/$\sqrt{3}$). The size measurements of aggregates were obtained with ImageJ using the greatest cross-area length of individual aggregates from top views. The software used for the data analysis was MICROSOFT EXCEL.

### Zeta potential and pH measurements

The Zeta potential of bacteria was measured on a Zetasizer Nano Serie 200. All bacterial samples were centrifuged and re-suspended in either 0.015% or 0.5% NaCl aqueous solutions (*S. aureus* and *P. aeruginosa* were centrifuged twice at 10,000 rpm for 10 min; *E. hirae* was centrifuged once at 10,000 rpm for 30 min). For *S. aureus*, the surface charge for each pH value was measured in three independent replicates. For each replicate, three sequences of 10 measurements were performed at each pH value. The charge for one replicate was obtained by averaging the measurements from all three sequences. The final charge was obtained by averaging the charge obtained for the three replicates. For *P. aeruginosa* and *E. hirae*, the charge of three technical replicates (three sequences of 10 measurements) were averaged for each pH value. The pH of each bacterial dispersions was measured with a VWR Scientific digital pH temperature meter (model 8015).

### SEM imaging

The *S. aureus* cell aggregates formed in Nutrient broth (pH 7) were observed by SEM. A 2 mL aliquot of the overnight culture was centrifuged at 10,000 rpm for 10 min and the resulting pellet was re-suspended in 1 mL of a 0.5% glutaraldehyde PBS solution and held for 30 min at room temperature. The centrifugation was repeated a second time and the bacterial pellet was re-suspended in 1 mL of sterile MilliQ water. An aliquot of 100 µL of bacteria was then drop casted onto a previously isopropanol washed silicon wafer and air dried overnight. The following day, the cells were fixed for 1 h with 100 µL of a 0.1% glutaraldehyde solution followed by an additional 1 h fixation with 100 µL of a 0.5% glutaraldehyde solution. Finally, the specimens were dehydrated by adding ethanol in a graded series (70% for 6 min, 90% for 6 min, and 100% for 6 min). The wafers were left to air dry and coated with platinum with a Cressington sputter coater before SEM imaging using a JEOL 7500 HRSEM.

### Time-to-kill study

First, 5 solutions of 90 mL of Nutrient broth were pH adjusted to 3.1, 5.0, 6.5, 9.2 and 11.0. Each solution was then divided into 3 sets of 3 solutions (3 replicates) each containing 10 mL of pH-adjusted Nutrient broth. In the first set, no DDAC (MAQUAT 4450-E didecyldimethylammonium chloride, Pilot chemical) was added; this series consists of untreated controls. A two ppm (final concentration) of DDAC solution was pipetted into the second set. A 20 ppm (final concentration) of DDAC solution was pipetted into the third set. Then, an aliquot of 100 µL of the overnight culture is mixed into each tube to reach a final *S. aureus* concentration of approximately 6.7 log_10_CFU mL^−1^. The samples were incubated at room temperature and were sampled at specified time intervals: 30 s, 1, 3, 5 and 10 min. At the end of each exposure time, an aliquot of 100 µL of each bacterial dispersion was added into 9.9 mL of TAT neutralizer broth (General Laboratory Products) and vortexed for five seconds. The vials containing TAT broth were incubated at 35 °C, 50 rpm for 18 h (MaxQ4000 shaker, model 4342, Thermo Scientific). Viability was assessed by the presence or absence of turbidity developed post incubation. The controls were incubated at room temperature for 10 min and were sampled for microbial enumeration (neutralization in TAT broth followed by serial dilution in phosphate buffer and plating on TSA plates). The CFUs were counted after 24 h of incubation at 35 °C. The untreated controls showed no reduction in the cell population (see Table S3 in Supplements for CFUs), suggesting that pH alone did not significantly impact cell viability. TAT broth was previously shown to be an effective media to neutralize DDAC^21^.

### Reversibility study

*S. aureus* dispersions in water with NaCl and in Nutrient broth were prepared as described in the aggregation study section of the “Materials and methods”. For each aforementioned medium, two aliquots of 4 mL of *S. aureus* dispersions (OD_600nm_ = 0.3, pH ~ 7) were adjusted to pH 3 and pH 9 using HCl and NaOH, respectively. Then, the pH of the first bacterial dispersion was adjusted to pH 9 and the second dispersion was adjusted to pH 3. All samples were homogenized with gentle manual shake and optical microscope imaging was carried out after each pH adjustment. For each medium, the experiment was conducted in three independent experiments with reproducible results.

### Bacterial specificity study

Aliquots (4 mL) of overnight cultures of *H. hirae* and *P. aeruginosa* were pH-adjusted using either HCl or NaOH. A gentle manual shake was performed to homogenize each sample before optical imaging. Two independent experiments were performed for each pH value with reproducible results.

### XDLVO simulations

Our simulation model is based on an extended DLVO^13,16–19^ (XDLVO) model. It relies on the seminal model for studying the aggregation of colloids. In the DLVO model, two main interaction potential are considered: the *Van der Waals* interaction energy^13^ (VdW) and the electrostatic repulsion interaction^16^. The XDLVO model incorporated two additional interactions: an attractive hydrophobic interaction^13,17^ and repulsive steric interactions^13^. The energy landscape was computed and the energy minimum was extracted. From the value of the minimum of energy, the percentages of bacterial populations either in singlets or in aggregates were calculated^18,19^ (see the Supplementary information section for more details).

## Supplementary Information

Supplementary Information.

## Data Availability

All data are available from the corresponding author upon request.

## Code availability

All codes are available from the corresponding author upon request.
