# Supplementary material for: Physical mechanisms driving the reversible aggregation of Staphylococcus aureus and response to antimicrobials
Source: Sci Rep. 2021 Jul 22;11:15048. doi: 10.1038/s41598-021-94457-1 (PMC8298462; doi:10.1038/s41598-021-94457-1)
Supplement: Supplementary file 1 — Supplementary Information. [file 41598_2021_94457_MOESM1_ESM.pdf]

Supplementary information

Physical mechanisms driving the reversible aggregation of *Staphylococcus aureus* and response to antimicrobials.

Céline Burel[1]*, Rémi Dreyfus[1] and Laura Purevdorj-Gage[2]

[1]Complex Assemblies of Soft Matter Laboratory (COMPASS), IRL 3254, CNRS-Solvay-University of Pennsylvania, CRTB, Bristol, PA 19007, United States

[2]Solvay, Novecare CRTB, Bristol, PA 19007, United States

*Correspondence to: Celine Burel, COMPASS, SOLVAY, 350 George Patterson Boulevard, Bristol 19007, PA, USA. E-mail: celine.burel@solvay.com

Frequency and distribution of aggregates

To monitor changes in the evolution of frequency and distribution of aggregates in a sample population, the number of small ("N_{Agg48}": number of aggregates of size between 4 and 8 μm) and larger ("N_{Agg8}": number of aggregates of size above 8 μm) aggregates per 10^3 bacteria cells were also quantified across the same pH range. In each medium, significant evolution of both N_{Agg8} and N_{Agg48} were observed as pH decreased below pH_{Agg}. N_{Agg8} continuously increases until pH reached about 3.2 whereas N_{Agg48} reached a maximum at a less acidic pH. For all three media type, ~ 12 aggregates of size larger than 8 μm per 1,000 cells are observed at pH 3.2. The decrease in N_{Agg48} may mean that cellular aggregates are aggregating, thereby decreasing their number. Yet, N_{Agg8} does not strongly increases because many small aggregates may become one larger aggregate. Overall, the evolution of both N_{C8} and N_{Agg8} as compared to N_{C48} and N_{Agg48} confirms the merging

of the small aggregates into bigger ones at pH ≤ 3.2, 5 and 7, for water at low and high salt concentrations and for Nutrient broth, respectively.

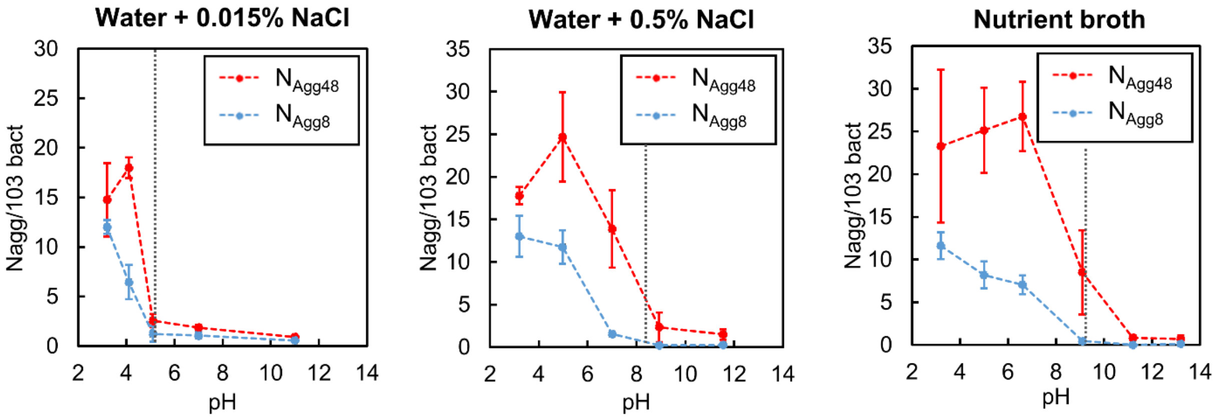

Fig. S1. Frequency and distribution of aggregates. Number of aggregates of size 4 to 8 µm (N_{Agg48}) and above 8 µm (N_{Agg8}) per 1,000 cells as a function of pH in water with 0.015% and 0.5 % NaCl and in Nutrient broth. The dashed line corresponds to pH_{Agg}.

Cell surface hydrophobicity assay

Cell hydrophobicity was measured by following the standard bacterial adherence to hydrocarbons (BATH) test in which the hydrophobicity is estimated by comparing the optical density of a bacterial suspension before and after mixing with hydrocarbons. The overnight culture was washed twice by centrifugation (Allegra 64 R Centrifuge) at 10,000 rpm for 10 min and the resulting pellets were re-suspended in either water or in water containing 0.5 % NaCl to an OD_{600nm} of 0.3. The bacterial suspensions were then pH adjusted with either HCl or NaOH. Sterile water was used as a negative control, and the optical density at 600 nm was measured as OD_0. Subsequently, a 2 mL cell suspension was mixed with a 1.2 mL of octane by mixing for 1 min (vortex), and the mixture was then left to phase separate for 10 min. The OD_{600nm} value of the aqueous phase was determined

as OD_1. The hydrophobicity percentages were calculated by the following equation: hydrophobicity (%) = $(1 - OD_1/OD_0) \times 100\%$. All experiments were executed only once.

The cell hydrophobicity was measured in the pH range of 3 to 13 in increments of 2 pH units. Fig. S2 summarizes the results obtained in all three media type. Fig. S2 (a) shows an optical microscope image (Leica SP8) of hydrophobic *S. aureus* adsorbed onto oil droplets in (i) water, (ii) water with 0.5% NaCl and (iii) in Nutrient broth at pH 7. *S. aureus* was found to be highly hydrophobic in both water and water containing NaCl (88 < Hydropbobicity (%) < 100, Fig. S2 (b) and (c)). The hydrophobicity of *S. aureus* in full broth could not be measured due to interferences from broth components (potentially due to adsorption of proteins on the oil droplets). Nevertheless, the possible interactions (adsorption, ionic interactions…) of the broth proteins with the *S. aureus* cells surface at different pH values and their consequences on the cell hydrophobicity were briefly explored. An aliquot of 10 ml of the overnight culture was pH adjusted first and then re-suspended in water containing 0.5 wt% NaCl with two wash steps to a final OD_{600nm} of 0.3. The results presented in Fig. S2 (c) do not show any impact of pH adjustment pre-centrifugation on the final bacterial hydrophobicity: *S. aureus* remained highly hydrophobic.

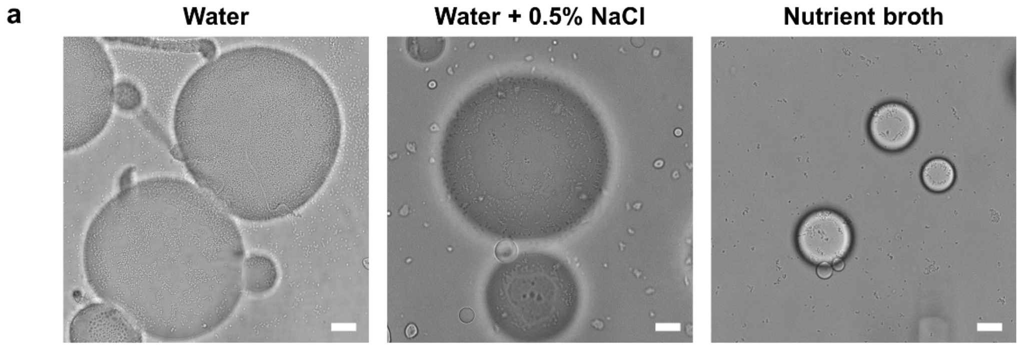

b

**pH**	3.0	5.2	7.0	8.7	11.1	12.8
**Hydrophobicity (%) in water**	100 +/- 12				87.9 +/- 12	100 +/- 12

**pH**	3.2	4.9	7.0	9.0	11.3	12.9
**Hydrophobicity (%) in water + 0.5% NaCl pH adjusted after centrifugation**	100 +/- 12					

**pH**	3.4	7.0	9.5
**Hydrophobicity (%) in water + 0.5% NaCl pH adjusted before centrifugation**	100 +/- 12		

Fig. S2. *S. aureus* cells are hydrophobic regardless of pH or salt concentration. a, Image of *S. aureus* cells adsorbed onto octane droplets at pH 7 when hydrophobicity test was performed in water, water with 0.5% NaCl and Nutrient broth. Each scale bar is 10 μm. **b**, Hydrophobicity of *S. aureus* in water and water with 0.5% NaCl - pH adjusted after and before centrifugation as a function of pH.

Modeling of *S. aureus* aggregation

The aggregation curves of the bacteria are modeled in two steps. In a first step, an extended DLVO[1] (XDLVO) model is used to determine the minimum of interaction energy between two bacterial cells, which–for the sake of simplicity- were assumed to be spherical. Once the minimum of

interaction energy was determined, the Smoluchowsky equation was used to compute the fraction of a singlet cell population. The two steps are described in the following sections.

The diameter of *S. aureus* was estimated from SEM images and was set to 675 nm (see GraphS1).

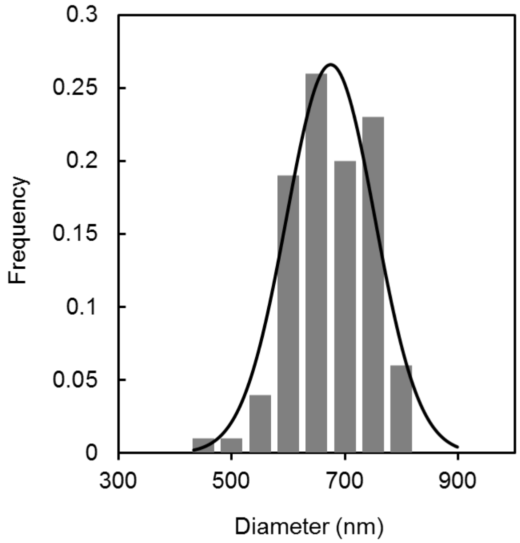

GraphS1: Histogram and Gaussian fit of the diameter of *S. aureus* cells as measured from SEM images.

<u>XDLVO:</u> The extended DLVO model is based on the seminal model for studying the aggregation of spherical colloids. In the DLVO model, two main interactions potential are considered: the Van der Waals interaction energy (VdW) and the electrostatic repulsion interaction. The expression for the VdW energy between two spheres of radius α, is shown in equation (1)[1]:

$$E_{VdW} = -\frac{A}{6}\left(\frac{2a^2}{D(4a+D)} + \frac{2a^2}{(2a+D)^2} + \ln\left(\frac{D(4a+D)}{(2a+D)^2}\right)\right), \quad (1)$$

where D is the distance between the two surfaces of the spherical bacteria and A is the Hamaker constant (in J). The expression for A for two colloids of index n_1 and electric susceptibility ε_1 is a medium of index n_2 and electric susceptibility ε_2 (see equation (2)):

$$A = \frac{3}{4}kT\left(\frac{(\varepsilon_1 - \varepsilon_2)^2}{(\varepsilon_1 + \varepsilon_2)^2}\right) + \frac{3I}{16\sqrt{2}}\frac{(n_1^2 - n_2^2)^2}{\left((n_1^2 + n_2^2)^{\frac{3}{2}}\right)}, \qquad (2)$$

where I is the ionization potential for the media (typically ~2.10^{-18}J)[1]. Here for bacteria in aqueous solutions, we took n_1 = 1.388[2], and n_2 = 1.33 for water, e_1= 1.9265 and e_2 = 1.7689. The weak index mismatch between bacteria and the aqueous solution results in a weak Hamaker constant ~9.33.10^{-22}J. The VdW interaction is attractive and often responsible for irreversible aggregation of colloids in solution.

Still, colloids like bacteria in solution do not always aggregate. Colloids often have charges on the surface which creates an electrostatic repulsive energy. The expression for the electrostatic repulsive energy is shown in equations (3) and (4)[3]:

$$E_{el} = 2\pi\varepsilon\psi_0^2 a\frac{2a}{2a+D}\exp(-\kappa D) \text{ for } \kappa a < 5, \qquad (3)$$

and

$$E_{el} = 2\pi\varepsilon\psi_0^2 a\ln(1+\exp(-\kappa D)) \text{ for } \kappa a > 5, \qquad (4)$$

Where ε is the electric susceptibility of the medium, ψ_0 is the surface potential of particles and κ is the Debye screening length. For z-z electrolyte, the expression of κ shown in equation (5) is:

$$\kappa = \left(\frac{\varepsilon kT}{2z^2e^2n_b}\right)^{-\frac{1}{2}}, \qquad (5)$$

where k is the Boltzman constant, T is the temperature and n_b is the number density of ions. In a DLVO model, the interaction energy between two colloids is basically the sum of these two interaction energies $E_{el} + E_{VdW}$. Depending on the parameter values, colloids can lower their energy by sitting in a potential well in the pair interaction.

For bacteria, other types of interaction potential can be added to these two interactions, thus forming the so-called extended DLVO (XDLVO)[1,4]. Several types of interactions can be added such as repulsive hydrophilic or attractive hydrophobic interactions[1,3,4] or sterical short-range interactions due to polymers or proteins located on the bacterial membrane[4]. Here we have added two types of interactions, (i) an attractive hydrophobic E_{hyd} based on the fact that we measured *S. aureus* to be hydrophobic and (ii) some short range sterical repulsive interaction E_{ster} based on the fact that we observed reversible aggregation of bacteria. The expression we choose for E_{hyd} is shown in equation (6)[1]:

$$E_{hyd} = -\alpha kT exp(-\frac{D}{\lambda}), \qquad (6)$$

where λ is a characteristic length usually of the order of 0.5 to 2 nm and a is the amplitude of the interaction at close contact in units of kT.

For the repulsive sterical interactions, we choose the expression shown in equation (7) for the energy:

$$E_{ster} = kT\frac{100aL^2}{\pi}\Gamma^{3/2}\exp(-\pi D/L), \qquad (7)$$

where L is the thickness of the polymer on the bacteria and Γ is the polymer density (m^{-2}) at the surface.

In the XDLVO model, the total pair interaction energy is the sum $E_{VdW} + E_{el} + E_{hyd} + E_{ster}$. For a certain set of parameter, we can compute the minimum of energy of the pair interaction energy E_{min}. Provided that E_{min} is known, we can calculate the fraction f of colloids/bacteria that will be suspended in solution. The way to calculate f has been described in details in[5,6]. To summarize, by considering the following equilibrium reaction:

$$A_1 + A_i \rightarrow A_{i+1},$$

where A_i is an aggregate of size *i,* one can construct a constant of reaction K (see equation (8)):

$$K = \frac{C_{A_{i+1}}}{C_1 C_{A_i}} \approx V \exp\left(-\frac{{}^{z}\!/_{2}\, E_{\min}}{kT} \right), \tag{8}$$

where V is the volume over which bacteria interact $V \sim 10^{-21} m^3$, z is the number of interacting neighbor in an aggregate z~6 and C_i the concentration of aggregates of size i. Once K is calculated, the fraction of bacteria remaining suspended in solution can be calculated (see equation (9))[6]:

$$f = \frac{1 + 2KC - \sqrt{1 + 4KC}}{2K^2C^2}, \tag{9}$$

where C is the total concentration of bacteria. Once f is calculated the percentage of bacteria in aggregates as shown in Fig. 1 (B) is 100(1-f).

We used this model to adjust these four unknown parameters, a, λ, Γ and L by fitting the aggregation curve for water with salt at 85 mM. The best fitting parameters are a = 36.5, λ= 1.8 nm, Γ= 4.10^{16}/m^2 and L = 1.61 nm. These results have order of magnitudes in agreement with those reported in the literature[4].

This model may find its use in predicting the effect of bacterial concentration and temperature on the aggregation in *S. aureus* in water with 0.5% salt (see Fig. S3 (c-d)). The concentration of bacteria had a large impact on aggregation. On the contrary, temperature seems to have a minor impact on aggregation within the time frame of our experiments. Based on these results, bacterial concentration is very likely to affect the time-to-kill values of QACs whereas temperature is not.

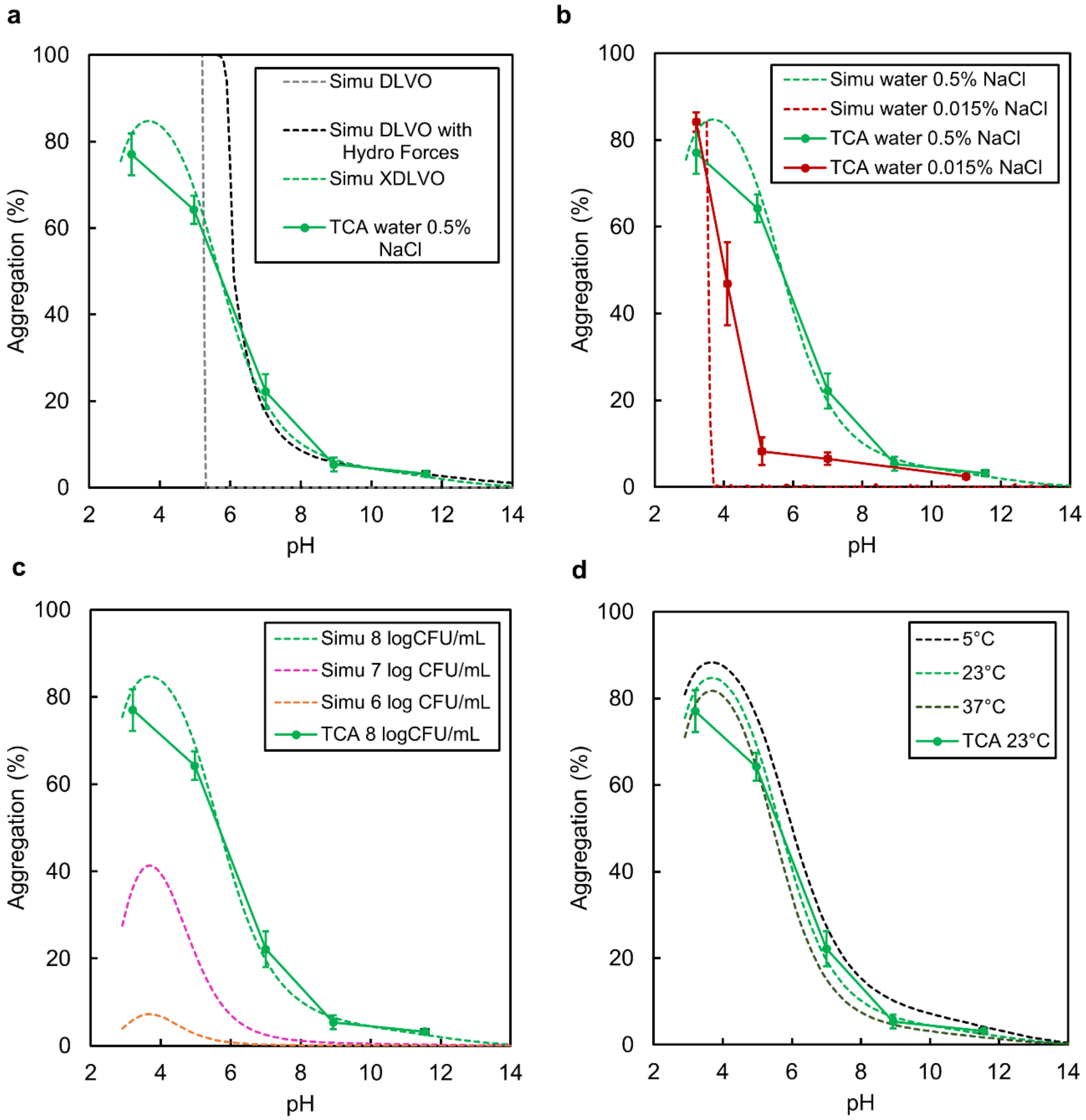

Fig. S3. Simulation of the percent of cell aggregated using the XDLVO model. Simulations (dotted lines) and experimental (solid lines) aggregation of *S. aureus* in **a**: water with 0.5% NaCl (the XDLVO model encompasses hydrophobic forces and short range repulsion), **b**: water with 0.015% or 0.5% NaCl, **c**: water with 0.5% NaCl as a function of the bacteria concentration, **d**: water containing 0.5% NaCl as a function of the temperature. Except stated otherwise, NaCl concentration is 0.5%, temperature is 23°C, the bacterial diameter is set to 675 nm and the bacterial concentration is set to 8 log_{10}CFU/mL.

Charge and size of proteins contained in Nutrient broth

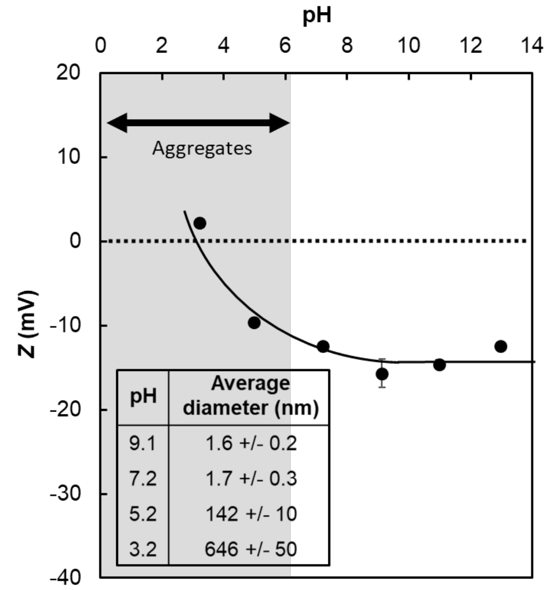

Fig. S4. Charge-mediated aggregation of the proteins in Nutrient broth as pH is decreased. Overall charge of proteins contained in Nutrient broth as a function of pH. Table in insert is the average diameter (in Number) of the proteins aggregates as a function of pH. As pH decreases the size of proteins increases, implicating a protein-protein aggregation. Concomitantly, as pH decreases, the charge of proteins decreases therefore suggesting a charge-mediated aggregation triggered by pH change. The black line is to provide visual guidance for readers. The aggregation and loss of charge of proteins could favor depletion and/or bridging of bacterial cells[7], thus promoting an increase in cell aggregation.

Regarding cell bridging, we hypothesize that proteins contained in Nutrient broth (peptone, beef extract) might become slightly positively charged at low pH due to protonation of both carboxylic and primary amine groups. By becoming more positively charged, the proteins might bridge negatively charged bacteria, hence enhance the cell aggregation[7].

Modeling of *S. aureus* aggregation: Effect of bacterial concentration in Nutrient broth

In Nutrient broth, where additional forces play a role in the aggregation of bacteria, the effect of bacteria concentration can be estimated by computing the minimum of energy. We can approximate the minimum of energy by solving numerically the equation (10) relating the fraction of singlets f to the constant of association K at the six different pH values used in the experiments (Fig. 1) for a known concentration C=10^8 bacteria /mL:

$$f(pH) = \frac{1+2K(pH)C - \sqrt{1+K(pH)C}}{2K(pH)^2C^2}, \tag{10}$$

Since the minimum of energy does not depend on concentration but on the surface-surface interactions between bacteria, once the constant K is numerically found, we can recalculate the fraction of singlet for different concentrations at the same six different values of pH. Results are shown in Fig. S5 for different bacteria concentration in Nutrient broth. The aggregation of the cells decreases with the decrease of the bacterial concentration.

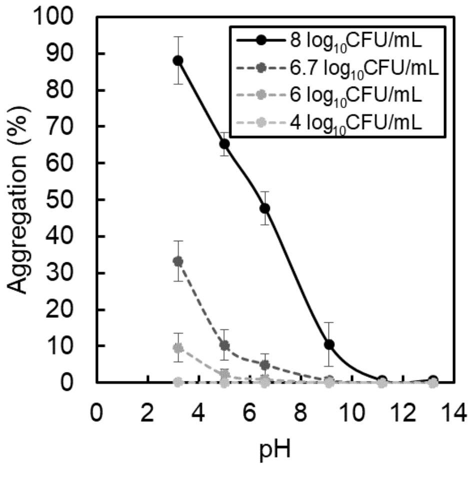

Fig. S5. Decrease of the percent of aggregated cell due to the decrease of the bacterial concentration in Nutrient broth. Simulations (dotted lines) and experimental (solid lines) aggregation of *S. aureus* in Nutrient broth at different concentrations of bacteria.

Since about 7 $\log_{10}$CFU/mL of *S. aureus* were not fully killed within 10 min with 20 ppm DDAC ($pH \leq 5$), we predict that *S. aureus* at 8 $\log_{10}$CFU/mL aggregated in larger proportion, will require more than 10 min for full kill with DDAC at or below 20 ppm for similar pH values. Similarly, time-to-kill in the order of 30 s might be achieved with bacteria at 8 $\log_{10}$CFU/mL in Nutrient broth at $pH > 11$ when the aggregation is below 10%.

Antimicrobial culture assay

<table>
<tr><th>pH</th><th>Contact time</th><th>Replicate 1</th><th>Replicate 2</th><th>Replicate 3</th></tr>
<tr><td rowspan="5">3.1</td><td>30 sec</td><td colspan="3" rowspan="5">Very turbid</td></tr>
<tr><td>1 min</td></tr>
<tr><td>3 min</td></tr>
<tr><td>5 min</td></tr>
<tr><td>10 min</td></tr>
<tr><td rowspan="5">5.0</td><td>30 sec</td><td colspan="3" rowspan="5">Very turbid</td></tr>
<tr><td>1 min</td></tr>
<tr><td>3 min</td></tr>
<tr><td>5 min</td></tr>
<tr><td>10 min</td></tr>
<tr><td rowspan="5">6.5</td><td>30 sec</td><td colspan="3" rowspan="5">Very turbid</td></tr>
<tr><td>1 min</td></tr>
<tr><td>3 min</td></tr>
<tr><td>5 min</td></tr>
<tr><td>10 min</td></tr>
<tr><td rowspan="5">9.2</td><td>30 sec</td><td colspan="3" rowspan="2">Very turbid</td></tr>
<tr><td>1 min</td></tr>
<tr><td>3 min</td><td>Turbid</td><td>Turbid</td><td>Clear</td></tr>
<tr><td>5 min</td><td colspan="3" rowspan="2">Clear</td></tr>
<tr><td>10 min</td></tr>
<tr><td rowspan="5">11.0</td><td>30 sec</td><td colspan="3">Very turbid</td></tr>
<tr><td>1 min</td><td>Very turbid</td><td>Turbid</td><td>Turbid</td></tr>
<tr><td>3 min</td><td colspan="3" rowspan="3">Clear</td></tr>
<tr><td>5 min</td></tr>
<tr><td>10 min</td></tr>
</table>

Table S1. Time-to-kill for 2 ppm of DDAC. The minimum time required to deactivate the entire *S. aureus* culture (in bold) upon 10 min exposure to [DDAC] = 2 ppm at different pH values.

<table>
<tr><th>pH</th><th>Contact time</th><th>Replicate 1</th><th>Replicate 2</th><th>Replicate 3</th></tr>
<tr><td rowspan="5">3.1</td><td>30 sec</td><td colspan="3" rowspan="4">Very turbid</td></tr>
<tr><td>1 min</td></tr>
<tr><td>3 min</td></tr>
<tr><td>5 min</td></tr>
<tr><td>10 min</td><td colspan="3">Turbid</td></tr>
<tr><td rowspan="5">5.0</td><td>30 sec</td><td colspan="3" rowspan="5">Very turbid</td></tr>
<tr><td>1 min</td></tr>
<tr><td>3 min</td></tr>
<tr><td>5 min</td></tr>
<tr><td>10 min</td></tr>
<tr><td rowspan="5">6.5</td><td>30 sec</td><td colspan="3" rowspan="2">Very turbid</td></tr>
<tr><td>1 min</td></tr>
<tr><td>3 min</td><td colspan="3" rowspan="3">Clear</td></tr>
<tr><td>5 min</td></tr>
<tr><td>10 min</td></tr>
<tr><td rowspan="5">9.2</td><td>30 sec</td><td colspan="3" rowspan="5">Clear</td></tr>
<tr><td>1 min</td></tr>
<tr><td>3 min</td></tr>
<tr><td>5 min</td></tr>
<tr><td>10 min</td></tr>
<tr><td rowspan="5">11.0</td><td>30 sec</td><td colspan="3" rowspan="5">Clear</td></tr>
<tr><td>1 min</td></tr>
<tr><td>3 min</td></tr>
<tr><td>5 min</td></tr>
<tr><td>10 min</td></tr>
</table>

Table S2. Time-to-kill for 20 ppm of DDAC. The minimum time required to deactivate the entire *S. aureus* culture (in bold) upon 10 min exposure to [DDAC] = 20 ppm at different pH values.

<table>
<tr><th>pH</th><th>Replicate 1</th><th>Replicate 2</th><th>Replicate 3</th><th>Average log_{10}CFU/mL</th></tr>
<tr><td>3.1</td><td colspan="3" rowspan="5">All solutions are very turbid</td><td>6.7</td></tr>
<tr><td>5.0</td><td>6.8</td></tr>
<tr><td>6.5</td><td>6.7</td></tr>
<tr><td>9.2</td><td>6.6</td></tr>
<tr><td>11.0</td><td>6.6</td></tr>
</table>

Table S3. Impact of pH on *S. aureus* viability for a contact time of 10 min. No decrease in viability is observed for the pH range 3 to 11.

References

1. Leckband, D., Israelachvili, J., Intermolecular forces in biology. *Q. Rev. Biophys.* **34,** 105–267 (2001).
2. Liu, P.Y. *et al.* Real-time measurement of single bacterium's refractive index using optofluidic immersion refractometry. *Procedia Eng*. **87,** 356 – 359 (2014).
3. Kovalchuk, N. M., Starov, V. M. Aggregation in colloidal suspensions: Effect of colloidal forces and hydrodynamic interactions, *Adv. in Colloid Interface Sci*. **179,** 99-106.
4. Dorobantu, L. S., Bhattacharjee, S., Foght, J. M., Gray, M. R. Analysis of Force Interactions between AFM Tips and Hydrophobic Bacteria Using DLVO Theory, L*angmuir* **25,** 6968–6976 (2009).
5. Dreyfus, R. *et al.* Simple quantitative model for the reversible association of DNA coated colloids. Phys. Rev. Lett. **102,** 048301 (2009).
6. Dreyfus, R. *et al.* Aggregation-disaggregation transition of DNA-coated colloids: Experiments and theory. Phys. Rev.E **81,** 041404 (2010).
7. Trunk, T., Khalil, H. S. and Leo, J. C. Bacterial autoaggregation. *AIMS Microbiol.* **4,** 140–164 (2018).
